# Cargo Recognition and Function of Selective Autophagy Receptors in Plants

**DOI:** 10.3390/ijms22031013

**Published:** 2021-01-20

**Authors:** Shuwei Luo, Xifeng Li, Yan Zhang, Yunting Fu, Baofang Fan, Cheng Zhu, Zhixiang Chen

**Affiliations:** 1 College of Life Sciences, China Jiliang University, Hangzhou 310018, China; luoshuwei@163.com (S.L.); 19a0902115@cjlu.edu.cn (X.L.); ting_2316156588@163.com (Y.F.); 2 Department of Landscape and Horticulture, Ecology College, Lishui University, Lishui 323000, China; yzhang@lsu.edu.cn; 3 Department of Botany and Plant Pathology, Purdue Center for Plant Biology, Purdue University, West Lafayette, IN 47907-2054, USA; bfan@purdue.edu

**Keywords:** autophagy, selective autophagy receptors, plants stress responses, NBR1, aggrephagy, ER-phagy, drought tolerance, plastid recycling

## Abstract

Autophagy is a major quality control system for degradation of unwanted or damaged cytoplasmic components to promote cellular homeostasis. Although non-selective bulk degradation of cytoplasm by autophagy plays a role during cellular response to nutrient deprivation, the broad roles of autophagy are primarily mediated by selective clearance of specifically targeted components. Selective autophagy relies on cargo receptors that recognize targeted components and recruit them to autophagosomes through interaction with lapidated autophagy-related protein 8 (ATG8) family proteins anchored in the membrane of the forming autophagosomes. In mammals and yeast, a large collection of selective autophagy receptors have been identified that mediate the selective autophagic degradation of organelles, aggregation-prone misfolded proteins and other unwanted or nonnative proteins. A substantial number of selective autophagy receptors have also been identified and functionally characterized in plants. Some of the autophagy receptors in plants are evolutionarily conserved with homologs in other types of organisms, while a majority of them are plant-specific or plant species-specific. Plant selective autophagy receptors mediate autophagic degradation of not only misfolded, nonactive and otherwise unwanted cellular components but also regulatory and signaling factors and play critical roles in plant responses to a broad spectrum of biotic and abiotic stresses. In this review, we summarize the research on selective autophagy in plants, with an emphasis on the cargo recognition and the biological functions of plant selective autophagy receptors.

## 1. Introduction

Autophagy is a highly conserved pathway in eukaryotes that recycles multiple cytoplasmic components under both normal and stress conditions such as starvation [1]. Induction of autophagy is initiated by the formation of an isolation membrane called phagophore that can extend to capture and sequester cytoplasmic components within a double-membrane vesicle termed autophagosome [2,3]. Mature autophagosomes can then fuse with the lysosomes or vacuoles for degradation of their cargo by resident hydrolases. The core machinery of autophagosome formation requires more than 40 largely conserved autophagy-related proteins (ATG). In vertebrates, these core autophagy components function in several physiologically continuous, but mechanistically distinct, steps and are organized into several functional complexes including: (i) the ULK (Unc-51 Like Autophagy Activating Kinase) complex with ULK1 and -2, ATG13, ATG101 and FIP200 (FAK Family Kinase-Interacting Protein of 200 kDa), (ii) the class III phosphoinositide 3-kinase (PI3K) complex I, with VPS34 (Vacuolar Protein Sorting 34), VPS15, Beclin1 and ATG14 for the nucleation and assembly of the initial phagophore membrane, (iii) the phosphatidylinositol-3-phosphate (PI3P)-binding ATG2A or -B and WIPI1–4 (WD Repeat Domain Phosphoinositide-Interacting Protein 1–4) complex, and iv) the two interrelated ubiquitin-like conjugation systems, ATG12–ATG5–ATG16 and ATG8–PE (phosphatidylethanolamine), which are required for the membrane elongation and expansion of the forming autophagosomes [4]. In addition, the ATG4 cysteine proteases process the precursors of ATG8 proteins for their lipidation and delipidation [4].

During cellular response to nutrient deprivation, autophagy usually involves non-selective uptake of cytoplasm into phagophores for bulk degradation of intracellular contents [5]. However, the broad roles of autophagy are primarily mediated by selective clearance of certain components [5]. In human cells, extensive studies have reported the selective autophagic degradation of aggregation-prone misfolded proteins and protein aggregates implicated in the pathology of various neurodegenerative diseases [5]. Furthermore, autophagy selectively degrades diverse organelles such as mitochondria, peroxisomes, lysosomes, endoplasmic reticulum (ER) and the nucleus, under various conditions [5]. Ubiquitin-like ATG8 plays a critical role in selective autophagy [6]. After attachment of the lipid PE to its carboxyl terminus through a conjugation pathway, ATG8 is both anchored in the membrane of autophagosomes and acts as a docking platform for the selective recruitment of cargos through a three-way interaction of selective autophagy receptors with both ATG8 and cargos [6]. Most selective autophagy receptors interact with membrane-anchored ATG8 through ATG8-interacting motifs (AIMs), which have the W/Y/F-X-X-L/I/V consensus core sequence [6]. AIMs of selective autophagy receptors bind a hydrophobic patch on ATG8 known as the AIM docking site [7]. A new class of selective autophagy receptors have been recently identified that interact with ATG8 through ubiquitin-interacting motif (UIM)-like sequences for high-affinity binding to an ATG8 interaction site different from the AIM docking site [8,9].

Autophagy has been extensively analyzed over the past two decades or so in Arabidopsis and, to a lesser extent, in other plants. Using genetic and molecular approaches, these extensive studies have established an important role of autophagy in almost all aspects of plant life, particularly in plant stress responses [10,11]. Autophagosome biogenesis and ATG gene expression are both induced under diverse abiotic stress conditions including nutrient starvation, heat, salt, drought and oxidative stresses [12,13,14,15,16,17]. Autophagy mutants and transgenic silencing lines display increased sensitivity to nutrient starvation and abiotic stresses when compared to wild-type plants [12,13,14,15,16,17]. In addition, plant mutants or transgenic silencing lines for autophagy are altered in response to virulent and avirulent biotrophic pathogens including pathogen-induced hypersensitive cell death [18,19,20,21,22,23]. Autophagy-deficient mutants are hypersusceptible to necrotrophic pathogens [19,24]. Furthermore, autophagy affects plant interaction with viral pathogens through regulation of antiviral RNA silencing, targeting degradation of viral proteins and other processes [20,25,26,27,28]. Autophagy also plays important roles in plant growth and development including root growth, leaf senescence, pollen and endosperm development [22,29,30,31,32].

**Figure 1:**
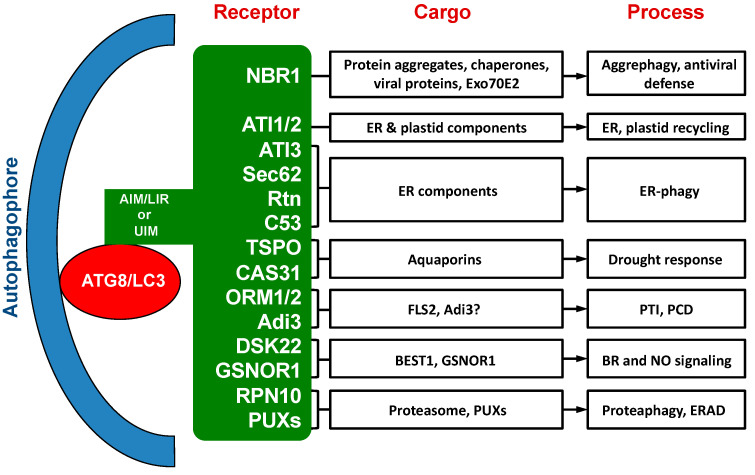
Cargo proteins and involved processes of selective autophagy receptors in plants.

Over the past ten years or so, a substantial number of selective autophagy receptors have been identified, characterized and functionally analyzed in plants [33,34] (Table 1; Figure 1). While a few of these autophagy receptors in plants are evolutionarily conserved with homologs in other types of organisms, most of them are plant-specific or even plant species-specific. The cargos recognized by these plant selective autophagy receptors include not only misfolded, nonactive and otherwise unwanted cellular components, but also regulatory and signaling factors [33,34]. Characterization of these plant autophagy receptors and their cargos have provided important new insights into the critical roles of autophagy in plant responses to a broad spectrum of biotic and abiotic stresses. Several recent reviews have covered selective autophagy in plants that also include discussion on well-studied selective autophagy receptors from plants [34,35,36]. In this review, we provide a comprehensive discussion on selective autophagy receptors from Arabidopsis and other plants including several that have just been recently reported. Functional characterization of selective autophagy receptors in plants is also advancing rapidly, with new discoveries of specific cellular components targeted by some of the selective autophagy receptors from plants. The review will present an in-depth and up-to-date analysis on the cargo recognition and biological functions of selective autophagy receptors in plant growth, development and stress responses.

## 2. NBR1 (Neighbor of BRCA1)

Among the identified autophagy receptors in plants, NBR1 has been most extensively characterized. Plant NBR1 is a structural homolog and functional hybrid of mammalian autophagy receptors NBR1 and p62 [51,52]. Both mammalian p62 and NBR1 proteins contain an N-terminal PB1 (Phox and Bem1p) domain, a ZZ-type zinc finger domain, an LC3-interacting region or LIR motif (also known as AIM motif in yeast and plants) and a C-terminal UBA (ubiquitin-associated) domain [53]. In addition, there is a highly conserved globular domain characterized by the presence of four highly conserved tryptophan residues in NBR1 but not in p62 [53]. Only metazoans contain both p62 and NBR1 homologs, while other eukaryotic organisms only have NBR homologs [53]. Plant NBR1 homologs lack the coiled coil domain of mammalian NBR1 but have two C-terminal UBA domains [37]. Model plant Arabidopsis contains a single gene encoding an NBR1 homolog, which, however, can homo-oligomerize through the N-terminal PB1 domain like p62 [37]. Only the C-terminal UBA domain of the two UBA domains of Arabidopsis NBR1 binds ubiquitin [37].

The biological functions of plant NBR1 have been analyzed through characterization of *nbr1* mutants or transgenic silencing lines. Arabidopsis *nbr1* knockout mutants are normal in growth and development under normal growth conditions. The *nbr1* mutants are also normal in general autophagy and in the selective clearance of peroxisomes, mitochondria, or the endoplasmic reticulum (ER) [16,54,55,56]. Plant NBR1 is not essential either for age- and darkness-induced senescence but may modulate growth or senescence under certain conditions such as short-day growth condition or under mineral deficiency [16,52,57]. The Arabidopsis *nbr1* mutants also respond normally to a necrotrophic pathogen [16]. However, loss of Arabidopsis *NBR1* gene function compromise plant tolerance to heat, oxidative, salt, and drought stresses [16,55]. The role of NBR1 in plant abiotic stress tolerance is mediated by selective autophagy based on its dependent on the interaction with ATG8 and is associated with the clearance of aggregation-prone misfolded proteins and protein aggregates [16] (Figure 1). In Arabidopsis, NBR1 also plays a role in resistance to the bacterial pathogen *Pseudomonas syringae* by suppressing the establishment of an aqueous extracellular space (“water-soaking”) [58]. More recent studies have further revealed important roles of plant NBR1 in the modulation of plant heat stress memory, plant–viral interaction and other stress-associated processes. These new roles of NBR1-mediated selective autophagy will be discussed in more detail here.

**Figure 2:**
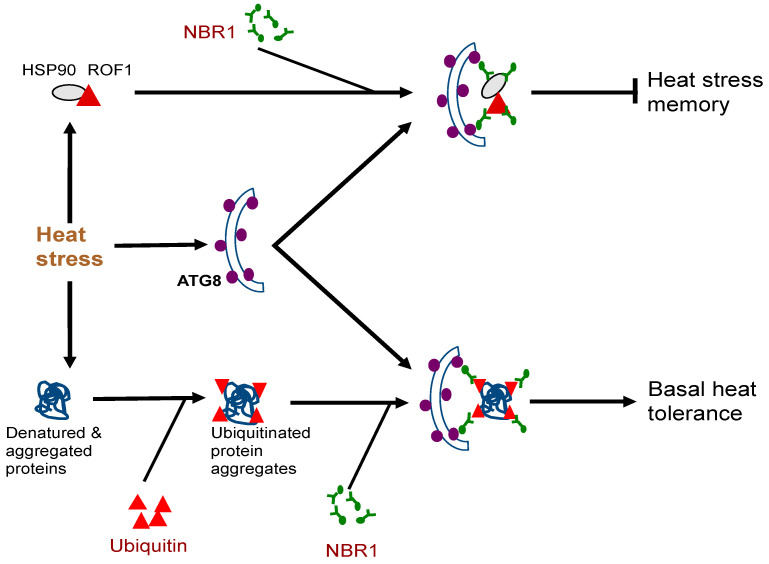
Complex roles of plant NBR1 (Neighbor of BRCA1) in plant heat stress responses. It mediates autophagic degradation of misfolded/denatured proteins or protein aggregates to mitigate heat-induced proteotoxicity to promote basal heat tolerance. NBR1-mediated selective autophagy also targets degradation of specific heat shock proteins (HSPs) such as HSP90 to reduce heat stress memory.

Very often, plants can be subjected repeatedly to a stress condition such as high temperature and need to balance between growth recovery and keeping stress memory for better survival when faced with a subsequent harsher stress [59]. Autophagy is induced in plants by moderate heat stress and targets a large number of proteins including specific heat shock proteins (HSPs) for degradation during the recovery phase after the end of heat stress, leading to reduced heat stress memory [59]. These target proteins include HSP90.1 and its interacting partner ROF1/AFKBP62 (rotamase FKBP 1), a plant homolog of mammalian FKBP4/FKBP52 [16,60]. The HSP90.1-ROF1 complex remains in the cytoplasm under normal conditions but binds heat shock transcription factor HSFA2 and translocates to the nucleus to activate heat-responsive gene expression following exposure to heat stress [61]. Degradation of HSP90.1 and ROF1 by NBR1-mediated selective autophagy attenuates HSFA2-dependent induction of HSP genes and represses the response to heat stress [60]. Indeed, the *nbr1* loss-of-function mutants is stronger in heat stress memory [60]. These results indicate that plant NBR1 plays complex roles in plant heat stress responses. It promotes basal heat tolerance mostly through autophagic degradation of misfolded/denatured proteins or protein aggregates to mitigate heat-induced proteotoxicity (Figure 2). After the end of heat stress, NBR1-mediated selective autophagy targets degradation of specific HSPs to reduce heat stress memory, probably to promote growth recovery but also downregulate acquired heat tolerance to a potential subsequent heat stress (Figure 2).

Autophagy plays an important role in plant–virus interactions. Previous studies have demonstrated that autophagy regulates virus-induced hypersensitive cell death and targets degradation of plant and viral proteins associated with dsRNA-induced RNA silencing [20,25]. More recent studies have revealed that NBR1-mediated selective autophagy targets degradation of specific viral proteins to suppress viral infection. In the study with *Cauliflower mosaic* virus (CaMV), it has been shown that NBR1-mediated selective autophagy targets nonassembled and virus particle-forming capsid proteins for degradation to restrict the establishment of CaMV infection [26]. To counter the antiviral defense mechanism, the CaMV-induced virus factory inclusions sequester the viral proteins and coordinate particle assembly and storage to protect capsid proteins against autophagic destruction [26]. NBR1 also targets the viral RNA silencing suppressor helper-component proteinase (HCpro), presumably in association with virus-induced RNA granules, to suppress accumulation of *Turnip mosaic* virus (TuMV), a positive-stranded RNA potyvirus [27]. Again, as counter defense mechanisms, several viral proteins have evolved the activity to antagonize NBR1-dependent autophagy. These results demonstrate the critical role of NBR1-mediated selective autophagy in plant antiviral defense and the potential viral strategies to evade and adapt autophagic processes for successful infection.

Recent studies have also demonstrated other cargos recognized and potentially targeted by NBR1 and further illustrate a broad role of the autophagy receptor in plant metabolism and stress responses. For example, Arabidopsis NBR1 is a selective receptor for Exo70E2 during autophagy in Arabidopsis [57]. Exo70E2 is a subunit of the exocyst complex, which directs the secretory vesicles of exocytosis from the Golgi complex to specific locations on the plasma membrane and to mediate their tethering and localization to the membrane immediately before fusion [62]. In Arabidopsis, there is a double-membrane organelle termed the exocyst-positive organelle (EXPO), which may be involved in mediating unconventional protein secretion in plants [63,64]. Exo70E2 is a marker for EXPO [63]. Upon induction of autophagy, Exo70E2-GFP positive EXPOs and autophagosome were colocalized and delivered to vacuoles for degradation in transgenic Arabidopsis plants [63]. Arabidopsis NBR1 specifically interacted and recruited Exo70E2 or its EXPO to ATG8-positive autophagosomes in a manner independent of its UBA domains [57]. Knockout of the NBR1 gene significantly reduced the vacuolar delivery of Exo70E2 or EXPO upon autophagic induction [57], supporting that the Arabidopsis NBR1-mediated selective autophagy pathway is involved in the vacuolar delivery of Exo70E2 or EXPO in plant autophagy.

Arabidopsis NBR1 also interacts with members of the plant-specific LSU (response to Low SUlfur) protein family, which are induced by sulfur (S) deficiency, suggesting a possible role of NBR1 in plant S nutrient responses [65]. Indeed, S deficiency induces autophagy and the transcription of *NBR1*. NBR1 overexpression alters plant gene expression in response to the low S conditions [66]. Furthermore, Arabidopsis seedlings overexpressing *NBR1* have significantly shorter roots than wild type when grown under S deficient conditions in the presence of TOR kinase inhibitors [66]. Arabidopsis NBR1 also interacts with three regulatory proteins of the abscisic acid (ABA) pathway (ABI3, ABI4 and ABI5) in planta [67]. NBR1 interaction with ABI5, but not ABI3 or ABI4, requires its UBA domain [67]. It is likely that ABI5, but not ABI3 or ABI4, requires ubiquitination prior to interaction with NBR1. It would be of interest to determine whether NBR1 binding of ABI3, ABI4 and ABI5 causes their autophagic degradation and affects their protein levels and ABA signaling in plants.

## 3. ATI1 and ATI2 (ATG8-Interacting 1 and 2)

Arabidopsis ATG8-interacting protein 1 and 2 (ATI1 and ATI2) were identified from yeast two-hybrid screen using ATG8f as bait [39]. The ATI1 and ATI2 are two closely related proteins that are unique to plants with 44% sequence identity and 67% similarity [39]. Sequence analysis indicated that ATI1 and ATI2 are transmembrane proteins with long N-terminal intrinsically disordered regions (IDRs) [68]. Although two putative AIMs were identified, a more recent study indicated that the N-terminal IDRs contain the functional AIM, which displays the disorder–order transition upon ATG8 binding based on nuclear magnetic resonance spectroscopy [68]. In addition, a sizable fraction of ATI2, but not ATI1, is phosphorylated in planta [68]. Under normal growth conditions, ATI1 and ATI2 are partially associated with the ER membrane network [39]. Upon exposure to carbon starvation, ATI1 and ATI2 become primarily associated with spherical compartments that are distinct from the ER bodies, Golgi, mitochondria, peroxisomes, and classical autophagosomes [39]. These compartments dynamically move along the ER network and ultimately traffic to the central vacuole [39]. Therefore, these ATI1- and ATI2-containing compartments may operate in selective turnover of specific proteins. Previously, it has been shown that ATI1 is involved in the recycling of chloroplast components under carbon starvation [40] (Figure 1). A more recent study has also demonstrated that ATI1 and ATI2 are components of a selective autophagy pathway that targets an ER-localized key component of posttranscriptional gene silencing (PTGS) [69] (Figure 1).

Chloroplasts are abundant in proteins and other molecules. Chloroplasts are dismantled during leaf senescence or under N or C starvation and their constituents are delivered by autophagosomes to vacuoles for degradation through several pathways [70]. Chloroplast stromal proteins such as Rubisco are delivered into the small double membrane structures called Rubisco-containing bodies (RCBs). The autophagic nature of RCBs is supported by the colocalization of RCBs with the ATG8 autophagosome marker [71]. Small starch granules (SSG) are delivered from chloroplasts to vacuoles by plastid-derived small spherical structures called SSG-like bodies in an autophagy-dependent manner [72]. The selective autophagy receptor ATI1 also mediates the delivery of chloroplast components to vacuoles for degradation [40]. Following carbon starvation, ATI1 is located on bodies associating with plastids. ATI1-plastid associated (ATI1-PS) bodies are distinct from the ER ATI bodies and are detected mainly in the periphery and inside of plastids in senescing cells undergoing plastid degradation. ATI1-PS bodies contain thylakoid membrane proteins, chlorophylls and other plastid components [40]. ATI1-PS bodies are released from chloroplasts into the cytosol independent of the autophagic machinery [40]. However, ATI1 on the plastid bodies interacts with ATG8f and their fusion with the central vacuole is dependent on functional autophagy [40]. These results indicate that ATI1 mediates a selective autophagy pathway with an important role in the autophagic plastid-to-vacuole trafficking of chloroplast components in senescence cells under carbon starvation.

RNA silencing is an evolutionarily conserved sequence-specific gene-inactivation system that also functions as an antiviral mechanism in plants and invertebrates [73]. To overcome the antiviral mechanism, viruses express silencing-suppressor proteins to counteract the host silencing-based antiviral process [73]. Plant ARGONAUTE1 (AGO1) is pivotal in RNA silencing and, therefore, is a major target of viral suppressors of RNA-silencing [69]. P0 from *Turnip yellows* virus (TuYV) is a suppressor of RNA silencing that can trigger AGO1 degradation via an autophagy-like process [69]. Both P0 and AGO1 are associated with the ER, loaded into ER-associated vesicles and are trafficked to the vacuole in an manner dependent on ATG5 and ATG7 [69]. ATI1 and ATI2 also associate with P0 and interact with AGO1 on the ER up to the vacuole. P0 expression induces degradation of ER-associated AGO1 that involves ATI1 and ATI2. In the mutant plants for ATI1 and ATI2, there is a significant increase in posttranscriptional gene silencing activity [69]. These results indicate that ATI1 and ATI2 are critical components of a selective autophagy pathway that promotes ER-associated AGO1 turnover to maintain proper levels of RNA silencing and is subjected to manipulation by the P0 suppressor of RNA silencing of TuYV as a counter defense mechanism.

## 4. ATI3s

Arabidopsis ATG8-interacting protein 3A (AT1G17780) was identified from yeast two-hybrid screens using ATG8a and ATG8f as baits [41]. ATI3A has two homologs, AT2G16575/ATI3B and AT1G73130/ATI3C from Arabidopsis [41]. The three proteins contain no known functional domain but all have a WxxL AIM/LIR motif at the C terminus that is required for interaction with ATG8. Analysis using BiFC showed that interaction of ATG8 and ATI3 proteins occurred under normal conditions but heat stress induced incorporation of the ATG8-ATI3 complexes into ATG8-labeled phagophores or autophagosomes based on increased formation of ATG8- and ATI3-labeled punctate structures in plant cells at high temperature [41]. Homologs of *Arabidopsis* ATI3 proteins are found only in dicots, but not in other organisms including monocots [41]. These ATI3 homologs are divergent in their amino acid sequences at the N-terminal region but share high sequence homology in the C-terminal region of approximately 100 amino acids and all contain a putative WxxL AIM/LIR motif at their C terminus [41].

Functional analysis using the mutants for the three genes indicated that these ATI3 proteins are dispensable for plant growth, development, age- and dark-induced senescence and salt tolerance [41]. On the other hand, single mutants for the three *ATI3* genes were all compromised in heat tolerance with *ati3a* mutant plants having the strongest phenotype [41]. The mutants for ATI3a, but not for ATI3b or ATI3c, were also compromised in resistance to the necrotrophic fungal pathogen *Botrytis cinereal* [41]. For examination of functional redundancy, *ati3a ati3b* double and *ati3a ati3b ati3c* triple mutants were also generated and were again found to be normal in growth and development under normal conditions but were compromised in both heat tolerance and disease resistance to extents similar to those of the *ati3a-1* single mutant [41]. These results indicated that among the three family members, ATI3a has a predominant role in plant heat tolerance and disease resistance [41]. The critical role of ATI3A in plant stress tolerance and disease resistance is dependent on its interaction with ATG8, implicating selective autophagy in the action of ATI3a [41].

ATI3A interacts with two close homologs of mammalian UBAC2 (Ubiquitin-associated domain-containing 2), a conserved protein implicated in endoplasmic reticulum (ER)-associated degradation (ERAD) [41]. Like the *ati3* mutants, mutants for the *UBAC2* genes were also compromised in both heat tolerance and resistance to *B. cinerea* [41]. As will be discussed in the next sections on selective autophagy receptors in ER-phagy, further analysis through overexpression of UBAC2 and characterization of *ati3* and *ubac2* mutants indicated that ATI3s play a critical role in plant heat tolerance and disease resistance at least in part by mediating selective autophagy of specific unknown ER components using ER-localized UBAC2 proteins as adaptors [41] (Figure 1). The evolutionarily conserved UBAC2 proteins in Arabidopsis also interact with an ER-localized, plant-specific protein, PICC, and promote accumulation of PMR4 callose synthase and pathogen-induced callose deposition [74]. Thus, despite the established role of UBAC2 in ERAD in mammalian cells [75,76], its plant homologs have novel roles in selective autophagy and plant immune responses.

## 5. ATI3s, Sec62, Reticulons and C53 in ER-Phagy

ER stress is defined as the accumulation of unfolded proteins in the ER, which can be induced by conditions such as heat or agents that cause ER stress, including tunicamycin and dithiothreitol [77]. ER stress-inducing agents activate autophagy in a manner dependent on inositol-requiring enzyme 1b (IRE1b) in Arabidopsis and activated autophagy delivers ER fragments to the vacuole for degradation, a process often referred to as ER-phagy [78,79]. ER-phagy is mediated by ER-phagy receptors [80]. To date, six ER-phagy receptors from mammals (FAM134B, RTN3L, CCPG1, SEC62, TEX264, and ATL3) and two from yeast (ATG39 and ATG40) have been reported [80]. These ER-phagy receptors are ER membrane proteins and contain at least one LIR or AIM for binding to autophagosomal LC3/GABARAP/ATG8 family proteins [80]. Some ER-phagy receptors can also interact with the most-upstream autophagy-initiation complex components including the Atg1 homologs ULK1 and ULK2, ATG13, ATG101, and FIP200 in mammals and ATG1, ATG13, ATG11, ATG17, ATG29, and ATG31 in yeast [80]. For example, mammalian CCPG1 and TEX264 ER-phagy receptors interact with FIP200, and ATG39 from yeast has a ATG11-binding region [80]. The interaction with the initiation complex was also found in other cargos of selective autophagy, such as mitochondria, intracellular bacteria, and p62 condensates [80].

Over the past several years, a number of proteins involved in ER cargo recognition for selective autophagy have also been identified and analyzed. As described earlier, Arabidopsis ATI3 proteins interact with ER-localized UBAC2 proteins [41]. Overexpression of UBAC2 induces recruitment of ATI3 proteins to phagophores or autophagosomes. Even though the *ati3* and *ubac2* mutants are fully competent in autophagy-dependent ER degradation under conditions of ER stress when using an ER lumenal marker for detection, they are significantly compromised in sensitivity to tunicamycin, an ER stress-inducing agent [41]. These results indicate that ATI3 and UBAC2 play an important role in plant stress responses by mediating selective autophagy of specific unknown ER components (Figure 1). Sec62 is a conserved component of the translocon complex that also acts as an ER-phagy receptor during the recovery phase of ER stress in mammals [80]. A recently reported study has demonstrated that the Arabidopsis Sec62 might also act as an ER-phagy receptor [42]. First, Arabidopsis Sec62, an ER-localized membrane protein, colocalizes with the autophagosome marker ATG8e in ring-like structures upon ER stress induction [42]. Second, Arabidopsis *sec62* mutants are impaired in vegetative growth and pollen development with reduced fertility [42]. The *sec62* mutants are also sensitive to tunicamycin-induced ER stress, whereas plants overexpressing *Sec62* display increased stress tolerance during the recovery phase of ER stress [42]. These results provide strong evidence that Arabidopsis Sec62 plays a pivotal role in plant development and ER-phagy (Figure 1).

Homologs of another evolutionarily conserved ER protein have also been recently shown to act as ER-phagy receptors. Reticulons (Rtn) proteins reside predominantly in the ER, primarily playing a role in promoting membrane curvature and, in some cases, acting as autophagy receptors for selective ER turnover [81]. Very recently, it has been demonstrated that maize Rtn1 and Rtn2 play an important role in ER homeostasis and autophagic flux in endosperm aleurone cells, which accumulate lipid droplets and synthesize storage protein accretions metabolized during germination [43]. Both Rtn1 and Rtn2 bind ATG8a through the four AIMs at the C-terminus, cytoplasmic loop, and within the transmembrane segments [43]. Binding of Rtn2 to ATG8 is enhanced by ER stress [43]. Mutants for Rtn1 and Rtn2 were normal in growth and generated viable progeny. The mutants have normal storage protein profiles and lipid droplet morphology in the developing aleurone cells [43]. However, unlike aleurone vacuoles from wild-type maize seeds, which were typically empty of other inclusions besides the protein accretions, the *rtn1* and *rtn2* mutants frequently contained cytoplasmic material, including ER, mitochondria, and other organelles, in the vacuoles from the aleurone cells [43]. The increased accumulation of cytoplasmic material in the aleurone vacuoles of the *rtn1* and *rtn2* mutants was dependent on functional autophagy, indicating that the loss of Rtn2 leads to an increase in ATG8-mediated macroautophagy [43]. The increased macroautophagy in the *rtn2* mutant indicates that reticulons play a critical role of in the ER homeostasis and suppression of ER stress [43]. In the *rtn1* and *rtn2* mutants, bulk autophagy might attempt to compensate for impaired ER turnover caused the loss of Rtn-mediated ER-phagy with increased ER stress [43]. These results support the role of reticulons in ER-phagy and in the suppression of ER stress (Figure 1).

Most ER-phagy receptors are activated during starvation or stress conditions and act together to remodel the ER network to maintain proteostasis. How ER-phagy acts with the core ER quality control pathways is less known. Recently, it has been reported that a conserved cytosolic protein, C53, is specifically recruited to autophagosomes in an ER stress-dependent manner, in both plant and mammalian cells [44]. C53, also known as CDK5RAP3 and LZAP, was first identified as a binding protein of mammalian cyclin-dependent kinase 5 (CDK5) activator. C53 also interacts with other proteins involved in cell cycle, cell survival and tumorigenesis [82]. C53 interacts with ATG8 via a non-canonical shuffled ATG8 interacting motif (W/F/Y-X-X-L/I/V; sAIM) [44]. C53 recruitment to autophagosomes is activated by proteotoxic stress in the ER lumen [44]. C53 senses ER stress by forming a complex with the ER-associated ufmylation ligase UFL1 and its membrane adaptor DDRGK1 [44]. The C53/UFL1/DDRGK1 tripartite receptor complex is activated upon ribosome stalling during co-translational protein translocation and mediates autophagic degradation of internal or passenger proteins in the ER [44]. Unlike the core autophagy mutants *atg5* and *atg2*, Arabidopsis *c53* mutants were normal under carbon or nitrogen starvation conditions. However, the *c53* mutants were hypersensitive to phosphate starvation, which is known to trigger an ER stress response [44]. Likewise, the *c53* mutants displayed increased sensitivity to tunicamycin treatment, which induces ER stress [44]. Furthermore, mutants for ufmylation machinery, including *ufl1* and *ddrgk1*, were sensitive to tunicamycin treatment but normal in sensitivity to carbon and nitrogen starvation [44]. These results indicate that C53 links selective autophagy with ribosome-associated quality control in the ER (Figure 1).

## 6. TSPO and MtCAS31 in Drought Stress Responses

Arabidopsis TSPO (tryptophan-rich sensory protein) is a member of the TspO/MBR (mitochondrial benzodiazepine receptor homolog) domain-containing membrane proteins related to the bacterial outer membrane TspO and the mammalian mitochondrial 18 kDa Translocator Protein (18 kDa TSPO) [45]. Arabidopsis TSPO is localized in the ER and the Golgi stacks [83]. TSPO is mainly detected in dry seeds, but can be induced in vegetative tissues by osmotic or salt stress or abscisic acid treatment [45]. On the other hand, boosting tetrapyrrole biosynthesis enhances TSPO degradation, which is dependent on its ability to bind heme [45]. TSPO degradation was inhibited in the autophagy-defective *atg5* mutant and by inhibitors of type III phosphoinositide 3-kinases, which regulate autophagy in eukaryotic cells [45]. TSPO contains an AIM for binding ATG8 and mutation of the two Tyr residues in the AIM in AtTSPO did not affect heme binding but stabilized the protein in vivo [45]. These results indicate that downregulation of TSPO is through degradation by the active autophagy pathway, which may serve to scavenge heme during stress in plants.

TSPO also interacts with the plasma membrane aquaporin PIP2; 7 at the ER and Golgi membranes in planta [84]. The TSPO-PIP2; 7 interaction is dependent on binding of phosphoinositides (PIs) by the plant-specific N-terminal extension of TSPO. Expression of TSPO increased phospholipase C activity and depleted PI (4,5) P2 from the plasma membrane but enhanced enrichment in Golgi membranes. Overexpression of TSPO also reduced accumulation of overexpressed PIP2; 7 in the plasma membrane and abolished the membrane water permeability mediated by transgenic PIP2; 7 [84]. Inhibition of autophagy enhanced the stability of both TSPO and PIP2; 7, suggesting that the autophagic pathway is responsible for the degradation of the TSPO/PIP2; 7 complex [84] (Figure 1). Interestingly, plasma membrane-localized aquaporin proteins are also subjected to degradation in the vacuole through the endocytic/multivesicular body (MVB) pathway [85]. In *Arabidopsis*, salt stress induces the internalization of PIP2; 1 from the plasma membrane through endocytosis to the vacuolar lumen though MVBs in a manner that is dependent on clathrin, phosphatidylinositol 3-kinase (PI3K) and PI4K [85]. Therefore, the autophagosome and MVB pathways act coordinately in down-regulating the levels of aquaporin proteins at the plasma membrane to reduce water transport under abiotic stresses. Selective autophagy increases degradation of newly synthesized aquaporin proteins at the ER and Golgi apparatus to reduce their transport to the plasma membrane. The MVBs pathway promotes degradation of those aquaporin proteins already localized in the plasma membrane through increased endocytosis.

Interestingly, MtPIP2; 7, an aquaporin protein from *Medicago truncatula*, is also targeted for degradation by selective autophagy under drought stress [46]. MtCAS31 (cold acclimation-specific 31), a dehydrin and a positive regulator of drought response from *M. truncatula*, plays a key role in autophagic degradation of MtPIP2; 7 [46]. A GFP cleavage assay and treatment with an autophagy-specific inhibitor indicated that MtCAS31 undergoes autophagic degradation and that overexpression of MtCAS31 promotes autophagy under drought stress [46]. Furthermore, MtCAS31 interacts with both ATG8 through the AIM-like motif YXXXI and MtPIP2; 7, which functions as a negative regulator of drought response [46]. These results indicate that MtCAS31 facilitates the autophagic degradation of MtPIP2; 7 to decrease root hydraulic conductivity, thereby reducing water loss under drought stress [46] (Figure 1). Targeting of aquaporin proteins by multiple selective autophagy receptors and by both autophagy and endocytic pathways underscores the importance of downregulation of the water channel proteins in plant drought tolerance.

## 7. Tomato Adi3 and ORM1/2 in Immune Responses

Plants have developed a complex immune system to protect themselves from infection [86]. Upon recognition of pathogen-associated molecular patterns (PAMPs) by plant pattern-recognition receptors, early plant defense mechanisms are rapidly triggered, which include a burst of reactive oxygen species (ROS), activation of mitogen-activated protein kinases (MAPKs), increased callose deposition and defense gene expression. Pathogens can deliver effectors to plant cells to suppress PAMP-triggered immunity (PTI) but some of the effectors may be recognized by plant resistance (R) proteins and activate effector-triggered immunity (ETI), which is a strong defense response often manifested as hypersensitive responses associated with rapid programmed cell death (PCD) [86] and increased accumulation of salicylic acid (SA) in both local infected and distant uninfected tissues to establish systemic acquired resistance [87].

As discussed earlier, autophagy affects plant responses to virulent and avirulent biotrophic pathogens including pathogen-induced hypersensitive cell death. A number of selective autophagy receptors have so far been identified in plants that regulate various processes associated with plant immune responses. For example, NBR1-mediated selective autophagy limits the growth of *Pseudomonas syringae* by suppressing the establishment of an aqueous extracellular space (“water-soaking”) [58]. To counter the defense mechanisms, *Pseudomonas* employs the effector protein HopM1 to activate autophagy and proteasome degradation to promote its pathogenicity [58]. In tomato, the Ser/Thr AGC protein kinase Adi3 suppressed PCD, at least in part, through a mechanism involving autophagy [48,88,89]. Adi3 was initially identified through its interaction with the effector protein AvrPto from the tomato pathogen *P. syringae* and the host resistance protein Pto [90]. The interaction of Pto and AvrPto leads to PCD associated with the hypersensitive response and resistance to *P. syringae* [48,89,90,91]. Virus-induced silencing of Adi3 leads to spontaneous cell death on stems and leaves, reduced plant size and, ultimately, plant death [48]. Importantly, Adi3 interacts with ATG8h and this interaction is not dependent on the kinase activity status of Adi3 [48]. Silencing of genes involved in autophagy is known to cause runaway PCD [48]. Cosilencing *Adi3* with several autophagy genes also leads to enhanced cell death, supporting that Adi3 may be involved in autophagic regulation of PCD [48] (Figure 1).

A recent study has demonstrated that selective autophagy targets degradation of nonactivated Arabidopsis pattern recognition receptor FLS2 (FLAGELLIN-SENSING 2), an immune receptor kinase that recognizes bacterial flagellin for PTI activation [47]. Arabidopsis orosomucoid (ORM) proteins act as selective autophagy receptors to mediate the degradation of FLS2 [47]. Overexpression of Arabidopsis plants *ORM1* or *ORM2* greatly reduced FLS2 accumulation, almost abolished FLS2 signaling and rendered plants more susceptible to the bacterial pathogen *P. syringae* [47]. On the other hand, *ORM1/2* RNAi plants and *orm1* or *orm2* mutants have increased levels of FLS2 and elevated FLS2 signaling, and are more resistant to *P. syringae* [47]. Importantly, ORM proteins interact with both FLS2 and ATG8 [47]. Overexpression of *ORM1* or *ORM2* in autophagy defective mutants has little effect on FLS2 abundance as it was comparable to that in wild-type plants [47]. Moreover, overexpressing ORM1/2 derivatives that do not interact with ATG8 did not reduce FLS2 levels either in Arabidopsis plants [47]. Taken together, these results suggest that ORM proteins act as selective autophagy receptors that target the degradation of a plant immune receptor in order to maintain its homeostasis [47] (Figure 1).

## 8. DSK2 and GSNOR1 in Signaling

Plant hormones play vital roles in plant growth, development and stress responses. Plant hormone signaling plays critical roles in regulating autophagy and plant stress responses [92]. Autophagy can also regulate hormone biosynthesis and signaling [92]. Two recent studies have demonstrated that selective autophagy regulates plant stress responses through targeted degradation of DSK2 and GSNOR1 involved the signaling of brassinosteroids (BRs) and nitric oxide (NO), respectively (Figure 1). BRs are an important class of plant hormones with critical roles in plant growth, development plant stress responses such as extreme temperatures and drought [93]. Signaling of BRs is initiated at the cell surface upon recognition by plasma membrane-localized receptors BR-INSENSITIVE-1 (BRI1), and its homologs, BRI1-LIKE-1 (BRL1) and BRL3. The binding triggers interaction with BRI1-ASSOCIATED-KINASE-1 (BAK1) (or members from the SOMATICEMBRYOGENESIS-RECEPTOR-KINASE (SERK) family), leading to the transphosphorylation of their kinase domains [93]. Activation of receptor complexes activates a signaling cascade that leads to the accumulation of BRASSINAZOLERESISTANT-1 (BZR1) and BR-INSENSITIVE-EMS-SUPPRESSOR-1 (BES1) transcription factors in the nucleus, which control expression of BR-regulated genes [93]. BR-deficient or insensitive mutants are more tolerant to plant stress than wild-type plants, indicating a negative role of BRs in plant stress responses [49,94,95]. Autophagy modulates BR responses through degradation of BES1 and BZR1. Autophagic degradation of BES1 is mediated by the autophagy receptor DSK2 (dominant suppressor of KAR2), which also interacts with ATG8 [49]. The interaction of DSK2 with ATG8 is activated upon phosphorylation of DSK2 by the GSK3-like kinase BIN2, a negative regulator in the BR pathway [49]. Phosphorylation of DSK2 and turnover of BES1 integrates BR and autophagy pathways to achieve balances between growth and stress responses [49,93]. Similarly, carbon starvation leads to TOR inactivation, autophagy induction, and degradation of BZR1 transcription factor in the BR pathway [96]. By targeting the degradation of both BES1 and BZR1 transcription factors under stress conditions, autophagy helps modulate BR-promoted growth to promote stress responses (Figure 1).

NO regulates diverse biological processes in plants, such as germination, root development, stomatal closing, abiotic stress and defense responses [97]. It acts mainly through redox-based S-nitrosylation of specific Cys residues of target proteins [98]. The S-nitrosylation is modulated by the intracellular level of *S*-nitrosoglutathione (GSNO), a major bioactive NO species. GSNO is degraded by the GSNO reductase (GSNOR), a highly conserved master regulator of NO signaling [50]. It has been demonstrated that the Arabidopsis GSNOR is regulated by selective authophagy during hypoxia responses [50]. S-nitrosylation of GSNOR1 at Cys-10 results in conformational changes, exposing its AIM for binding by autophagy machinery [50]. Upon binding by ATG8, GSNOR1 is recruited into the autophagosome and selectveily degraded through autophagy in an AIM-dependent manner, leading to an increased NO levels [50]. Physiologically, increased NO level as a result of the S-nitrosylation-induced selective autophagy of GSNOR1 promotes seed germination under low oxygen conditions as part of plant hypoxia responses. These results establish a unique mechanism by which S-nitrosylation induces selective autophagy of GSNOR1 to establish a molecular link between NO signaling and autophagy [50] (Figure 1).

## 9. RPN10 and UIM-Containing Autophagy Receptors

In eukaryotes, the ubiquitin–proteasome system (UPS) and autophagy are two primary pathways for degradation of cellular constituents [99]. Generally, the UPS is responsible for degrading short-lived proteins and autophagy is primarily responsible for the removal of long-lived proteins, organelles and large protein complexes and aggregates [99]. In the UPS, a 19S regulatory particle identifies appropriate substrates and translocates them into the lumen of the 20S core protease, which contains peptidase active sites, for subsequent breakdown [100]. In *Arabidopsis*, nitrogen starvation and chemical or genetic inhibition of the proteasome induce degradation of the 26S proteasome by autophagy, which is referred to as proteaphagy [9]. There are distinct pathways for proteaphagy. While nitrogen starvation induces nonselective proteaphagy, proteasome inhibition leads to selective proteasome degradation, which requires the proteasome subunit RPN10 as a specific receptor through its concurrent interactions with ubiquitylated proteasome subunits/targets and lipidated ATG8 residing on the phagophore [9]. Unlike other autophagy receptors, RPN10 does not contain the canonical AIM but has three ubiquitin-interacting motifs (UIMs) [9]. The UIM1 in RPN10 is responsible for binding ubiquitylated proteasomes and UIM2 for binding ATG8, thereby forming a stable tripartite complex to be engulfed in autophagosomes for delivery to the vacuole and degradation [9] (Figure 1).

The well-defined AIM of other autophagy receptors recognizes a hydrophobic patch on ATG8 known as the LIR/AIM docking site (LDS). The discovery that Arabidopsis RPN10 uses its UIM2 sequence for binding ATG8 instead of the AIM indicates that ATG8 has a distinct surface other than the canonical LDS for this interaction. Indeed, the UIM2 from RPN10 binds ATG8 at an alternative ATG8 interaction site called the UIM docking site (UDS) [8]. Assays with UIM-containing candidate proteins and unbiased yeast two-hybrid screens identified a large number of UIM-based ATG8 interactors in plants, yeast, and humans [8]. These identified UIM-based ATG8 interactors include some members of the PLANT UBIQUITIN REGULATORY X (PUX) protein family from Arabidopsis [8]. Four members from the Arabidopsis PUX family (PUX7, PUX8, PUX9 and PUX13) act redundantly as receptors for autophagic degradation of CDC48 ATPase [8]. CDC48 is a homohexameric complex assembled as a ring and plays a role in diverse cellular processes including protein quality control by using ATP hydrolysis to extract polypeptides from protein complexes or membranes for retrograde transport from the ER lumen during ER-associated protein degradation (ERAD) for eventual turnover in the cytosol [8]. Thus, identification of this new class of UIM-containing autophagy adaptors and receptors greatly expands the reach of selective autophagy (Figure 1).

## 10. Summary and Prospect

Over the past ten years, major progress has been made in the identification and functional characterization of selective autophagy receptors in plants. These selective autophagy receptors mediate autophagic degradation of diverse molecules including not only unwanted and potentially toxic or harmful cytoplasmic components but also important factors in hormone and stress signaling and RNA silencing. As a result, analysis of these selective autophagy receptors and associated autophagy pathways have provided important new insights into the dynamic and intricate networks of plant responses during nutrient starvation or under different biotic and abiotic stress conditions. However, the research on plant selective autophagy is still at relatively early stages and our understanding of the broad roles and underlying mechanisms of selective autophagy pathways in plants is still very limited. First, the number of selective autophagy receptors that have been identified in plants is still relatively small when compared to that in mammals. In particular, there is little information on the selective autophagy receptors that mediate autophagic degradation of important plant cell organelles such as mitochondria and peroxisomes. Previously, Arabidopsis ATG11 has been shown to play an important role in general autophagy and senescence-induced mitophagy [101,102]. However, it remains to be determined whether ATG11 is responsible for the recognition of mitochondria in mitophagy. Both p62 and NBR1 are required for pexophagy in mammalian cells but Arabidopsis NBR1 is not essential for the selective clearance of peroxisome, mitochondria or the ER [16,54,56]. Second, even for many of those selective autophagy receptors that have been functionally analyzed in plants, more in-depth research is still necessary for a better understanding of their roles, dynamic action mechanisms and regulation. Third, autophagy is one of several major degradation pathways in eukaryotic cells. These pathways also include the 26S proteasome system and the clathrin-mediated endocytosis pathway that target degradation of cellular components in the vacuole. Extensive research has been reported in animals on the crosstalk and coordination among these degradation pathways in promoting cellular homeostasis but relatively little is known about the coordinative nature of autophagy with other degradative systems in plant cells. A better knowledge about the broad and complex roles, action mechanisms and regulation of selective autophagy receptors and mediated autophagic pathways will provide new important insights into the molecular basis of plant growth, development and responses to biotic and abiotic stresses.

## Figures and Tables

**Table 1:** Interacting ATG8 (autophagy-related protein 8) isoforms and recognition motifs of selective autophagy from plants.

Receptor	Interacting ATG8 Isoform	Recognition Motif	References
NBR1	ATG8s	AIM	[16,37]
ATI1/2	ATG8f, ATG8h	AIM	[38,39,40]
ATI3s	ATG8a, ATG8f	AIM	[41]
Sec62	ATG8e	AIM	[42]
ZmRtn1/2	ZmATG8a	AIM	[43]
C53	ATG8a–g, ATG8i	AIM	[44]
TSPO	ATG8e	AIM	[45]
MtCAS31	MtATG8a	AIM	[46]
ORM1/2	ATG8a, ATG8d, ATG8e, ATG8i	AIM	[47]
SlAdi3	SlATG8h	AIM	[48]
DSK2	ATG8e	AIM	[49]
GSNOR1	ATG8s	AIM	[50]
RPN10	ATGa, ATG8e, ATG8f, ATG8i	UIM	[8,9]
PUXs	ATG8a, ATG8e	UIM	[8]
